# Projecting the Global Distribution of the Emerging Amphibian Fungal Pathogen, *Batrachochytrium dendrobatidis*, Based on IPCC Climate Futures

**DOI:** 10.1371/journal.pone.0160746

**Published:** 2016-08-11

**Authors:** Gisselle Yang Xie, Deanna H. Olson, Andrew R. Blaustein

**Affiliations:** 1 Department of Integrative Biology, Oregon State University, Corvallis, Oregon, United States of America; 2 United States Forest Service, Pacific Northwest Research Station, Corvallis, Oregon, United States of America; Universitat Trier, GERMANY

## Abstract

Projected changes in climate conditions are emerging as significant risk factors to numerous species, affecting habitat conditions and community interactions. Projections suggest species range shifts in response to climate change modifying environmental suitability and is supported by observational evidence. Both pathogens and their hosts can shift ranges with climate change. We consider how climate change may influence the distribution of the emerging infectious amphibian chytrid fungus, *Batrachochytrium dendrobatidis* (*Bd*), a pathogen associated with worldwide amphibian population losses. Using an expanded global *Bd* database and a novel modeling approach, we examined a broad set of climate metrics to model the *Bd*-climate niche globally and regionally, then project how climate change may influence *Bd* distributions. Previous research showed that *Bd* distribution is dependent on climatic variables, in particular temperature. We trained a machine-learning model (random forest) with the most comprehensive global compilation of *Bd* sampling records (~5,000 site-level records, mid-2014 summary), including 13 climatic variables. We projected future *Bd* environmental suitability under IPCC scenarios. The learning model was trained with combined worldwide data (non-region specific) and also separately per region (region-specific). One goal of our study was to estimate of how *Bd* spatial risks may change under climate change based on the best available data. Our models supported differences in *Bd-*climate relationships among geographic regions. We projected that *Bd* ranges will shift into higher latitudes and altitudes due to increased environmental suitability in those regions under predicted climate change. Specifically, our model showed a broad expansion of areas environmentally suitable for establishment of *Bd* on amphibian hosts in the temperate zones of the Northern Hemisphere. Our projections are useful for the development of monitoring designs in these areas, especially for sensitive species and those vulnerable to multiple threats.

## Introduction

Climate change represents one of the greatest challenges to biodiversity because it may compromise the integrity and services of ecosystems worldwide. Changes in climate are affecting species interactions and habitat conditions. For example, climate conditions can alter the phenology of plant-animal interactions, predator-prey interactions, and disease dynamics [1]. Many species, including infectious agents and vectors, are already shifting their ranges to different latitudes and elevations as suitable habitat conditions change [1–4]. Climate-induced range shifts and species movements potentially affect host-pathogen interactions in complex ways [1] and may bring hosts into contact with novel diseases.

The emerging infectious amphibian chytrid fungus, *Batrachochytrium dendrobatidis* (hereafter “*Bd*”), causes chytridiomycosis which may be responsible for the most spectacular loss of vertebrate biodiversity due to disease in recorded history [5]. *Bd* is found on every continent where amphibians exist, infecting close to 700 amphibian species globally [6,7] and is implicated in worldwide amphibian population declines [8–12]. Yet, amphibian species and population vulnerability to *Bd* infection and developing chytridiomycosis varies (e.g., [13–17]). Also, *Bd* strain differences, potential strain hybridization, and strain attenuation account for differences in virulence [18–20]. For susceptible species, development of chytridiomycosis may cause excessive skin shedding, lethargy, loss of righting reflex, and eventually mortality in adults; in tadpoles *Bd* may adversely affect mouthpart structures, leading to malformations and altered feeding [5,8,9,21]. Further complicating *Bd*-host interactions are interacting environmental factors, including climate associations [6].

Given its apparent host non-specificity, many more amphibian species are likely to become suitable hosts if *Bd* emerges in their regions [22]. It is hypothesized that the emergence of *Bd* has been due to both spatial spread into naïve regions and enzootic states shifting to epizootic with environmental change, such as change in climate, which may induce change in host-pathogen interactions by decreasing host fitness or increasing pathogen virulence [23,24]. Moreover, habitat change and alteration with projected shifts in climate may affect amphibian host ranges [25–27], the range of *Bd* (e.g., *Bd* climate associations [6,22,28,29]), and amphibian-*Bd* dynamics, including spread to novel hosts. This scenario is further complicated as climate may affect habitat continuity for both *Bd* and host species, with habitat fragmentation influencing the capacity of organisms to respond to potential climate changes and many amphibians having naturally patchy distributions [30]. Independently, habitat loss and degradation are chief contributors to global amphibian declines with disease being an additional key contributing factor for some species [8]; hence climate change may interact and potentially alter these dynamic processes [30]. Also, we currently lack a full understanding of *Bd* transmission vectors, although free-living aquatic zoospores suggests *Bd* can move with water movements [31] and human-mediated transmission has been implicated (e.g., [32,33]), further complicating projections of *Bd* distributions contingent on globalization activities.

Currently, the spatially heterogeneous distribution of *Bd* may be due to its continuing spread in naïve regions [6], a conservation concern for native biodiversity. To inform preventative management and resource prioritization, correlative species distribution models (SDMs) have been used to characterize environmental suitability and the potential range for *Bd* [6,22]. SDMs are typically applied where the range of the target species, which is often free-living, is expected to be mainly regulated by environmental variables (e.g., climate, biome, habitat type [22]), as opposed to pathogens whose immediate environment is the product of both the environment and host physiology. For many pathogens, this may be inappropriate if host environments and the free-living portions of the pathogen lifecycle are insulated from external environmental conditions, which is especially true for internal, directly transmitted pathogens of endothermic hosts [22]. For *Bd*, amphibian hosts are ectothermic and there is an environmental zoospore stage for transmission [28,31,34]. As a result, *Bd* is directly subject to the effects of environmental variables, in particular temperature and moisture [22,28,29,31]. Furthermore, recent field research has revealed that *Bd* occupancy in the environment may not depend on the presence of amphibian hosts in the same locale which provides further support in using SDMs to project future *Bd* distribution without necessarily modeling changes in host distributions simultaneously [35]. Therefore, SDMs may be a valuable tool to evaluate environmental suitability for *Bd* and allow managers to identify areas suitable for the establishment of *Bd* [22]. Field observations of seasonal and altitudinal differences in *Bd* outbreak patterns have long suggested the climatic dependency of *Bd* [23,34,36,37]. While we should take care in interpreting the results of these SDM's, which are based on observational data and are fundamentally correlation-based, these observations have been supported by laboratory experiments [18–20]. Previous SDMs also have shown that both chytridiomycosis and *Bd* distribution are strongly associated with climatic variables [6,22,35,38–41]. Currently, little is known of the comparative environmental thresholds of *Bd* life stages, such as free-living *Bd* zoospores versus host-infected *Bd*, therefore we do not differentiate them in this analysis.

Previous SDMs have predicted a range of environmental conditions suitable for the occurrence of *Bd* [38,40–43]. As a result, it was suggested that the emergence of *Bd* may be curbed by a general warming trend of the climate since the fungus prefers cool, moist habitats [44] and a previous SDM indeed suggested that anthropogenic climate change may reduce the geographic range of *Bd* [45], although this prediction was subsequently found to be inconsistent with some field observations [46]. Also, initially, climate change was suggested to be promoting *Bd* emergence at some locations [23]. It was hypothesized that at high-altitude sites in Central America, increasing cloud cover may have been moderating daily minimum and daily maximum temperatures to converge around a ‘‘chytrid thermal optimum” [23] (but see [47,48]). The ‘thermal optimum’ hypothesis is consistent with the laboratory observation that fluctuating temperatures slow *Bd* growth [49], but has been criticized for interpreting a spurious correlation between climate and *Bd* emergence as causal [48]. However, *Bd* outbreaks in the Neotropics were more common following high-temperature years [50], suggesting that climate change and the associated increased temperatures might actually promote *Bd* emergence [46]. These apparently contradictory results describing the *Bd*-climate relationship may arise from complex interactions of climatic variables in the field or regional heterogeneity in either *Bd* biology or host-pathogen dynamics. Hence, these results highlight the uncertainties in how environmental suitability for *Bd* and its potential distribution might be affected by climate change.

In this paper, we provide an estimation of the future spatial risks to *Bd* under IPCC projected scenarios of climate change based on a significantly improved *Bd* distribution model in terms of prediction accuracy over previous SDMs. We formulate region-specific and global predictive models based on gridded climate data of 0.5° x 0.5° resolution (13 metrics analyzed, S1 Table) with the most current and extensive global *Bd* detection data set (~5000 world data records of *Bd*-positive and -negative sites) and project future *Bd* distributions under several climate change scenarios. We construct SDMs for *Bd* using random forest models, one of the most accurate learning algorithms [51]. Random forests use both detection and non-detection data instead of presence-only methods such as MAXENT, which artificially creates “pseudo-absence observations” from background sampling to construct discriminative models. Random forest is ideal for data sets with many explanatory variables and complex higher-order interactions, and has been consistently shown to yield high-prediction performance in comparison to other methods [51–56]. Random forests also perform well in predicting the distribution of invasive species [54]. We project global regions where *Bd* may increase or decrease in climate-niche suitability. Our predictions may inform decisions for prioritization of amphibian conservation efforts, especially monitoring resources.

## Materials and Methods

### *Bd* occurrence data

*Bd* detected/not-detected data were obtained from the 2014 global update of the world *Bd* database maintained by the curators of *Bd*-maps.net [6,7]. All available records of wild-caught amphibian samples up to June 2014 were compiled into site-level data, with a site having a common latitude/longitude coordinate. This consisted of 5166 site-level records for sites with coordinate information of sufficient accuracy for spatial analysis. We did not sort sites by *Bd* strain because records in the *Bd*-maps database do not include this parameter; post-hoc regional correspondence with strain could be problematic if recent dispersal or human-mediated transmission has affected strain geographic distribution. The recently described salamander chytrid fungus *B*. *salamandrivorans* (*Bsal*) differs from *Bd* genetically and morphologically; it is unknown if the *Bd*-maps database inadvertently includes *Bsal* data, although this likely has been minimized since the widespread use of *Bd*-specific qPCR methods [57], and differing host species [58]. *Bsal* has not yet been detected in the wild outside Europe. A web portal is in development to distinguish *Bd* and *Bsal* records (amphibiandisease.org).

Upon examination of the data, we found 129 site records with coordinates that duplicated another site. These duplicated sites often included both *Bd*-positive and -negative records; therefore we scored a site as *Bd*-positive as long as one observation showed *Bd* detection. Due to the spatial resolution of global climate data, our explanatory variables were extracted from a grid with a resolution of 0.5 degrees latitude and longitude. Consequently, 56 sites had the same explanatory information because they were clustered within cells on the grid, essentially duplicating each other. For the purpose of our analysis, we averaged the longitude and latitude, respectively, of these sites clustered within the same grid cell and used the averages as a new coordinate, and designated this new coordinate as *Bd*-positive as long as one record demonstrated *Bd* detection. We believe that these procedures have the effects of clarifying the relationship between response and explanatory variables by removing inconsistent classification at the same sites and also reducing clustering. A final total of 4967 sites (grid cells) were used in the analysis (S1 Fig). It should be noted that samples taken at these sites may be from animals with various *Bd* zoospore loads. Potential biases of the dataset are that sampling for *Bd* has not been randomly conducted, our *Bd* sample size varied among regions which may affect power of our analyses and hence increase uncertainty of our findings for regions with lower sample sizes, *Bd*-negative data may be under-reported, and *Bd*-negative data do not necessarily indicate that *Bd* is absent from a site, for example if sampling was insufficient to detect it at low prevalence. Consequently our models may be less sensitive for sites with low *Bd* prevalence, reflecting patterns for places with higher prevalence. Nevertheless, globally and in regions where our sample sizes were large, our comprehensive dataset enhances the power of such broad landscape-scale analyses to provide insights regarding climate-associated projections, and may aid development of hypotheses for further analysis using downscaled data from more rigorous sampling methods.

### Model formulation

We examined the associations between *Bd* occurrence at a site and both climatic and ecological variables. We focused on climatic variables, and included 13 temperature and precipitation metrics (S1 Table), which have been shown to perform well in previous *Bd* environmental suitability analyses [6,22,38,40–43]. Additionally, we included elevation [59], biome type [60], amphibian species richness [8], and whether an enigmatic amphibian decline was reported at the site [8], since these factors have been shown to correlate with *Bd* occurrence [6]. Richness was included as a parameter of the model to address the likelihood of more and diverse carriers to retain the fungus at sites. Climate variables were extracted per coordinate from grids with a resolution of 0.5 degrees latitude and longitude, available from the Climatic Research Unit at University of East Anglia (Norwich, UK, www.cru.uea.ac.uk) for the years 2000–2010 and averaged over the period. Even if samples at a site may have been collected outside of this time span, we believe that these averaged climate metrics would be able to represent “typical” climate at a site and reflect any current climate associations with *Bd* occurrence. The correlative nature of SDMs is important to highlight: if a site was sampled for *Bd* prior to 2000–2010, we cannot make the inference that there is a causal link between *Bd* occurrence at that site and the climate during 2000–2010. Although climate conditions might still be helpful in predicting *Bd* occurrence on the basis of correlations alone, caution must be exercised in using performance of these SDMs to support causal links between climate and *Bd* occurrence at a site. It is possible that *Bd* occurrence may in fact be determined by some unmeasured variables highly correlated with climate.

**Fig 1:**
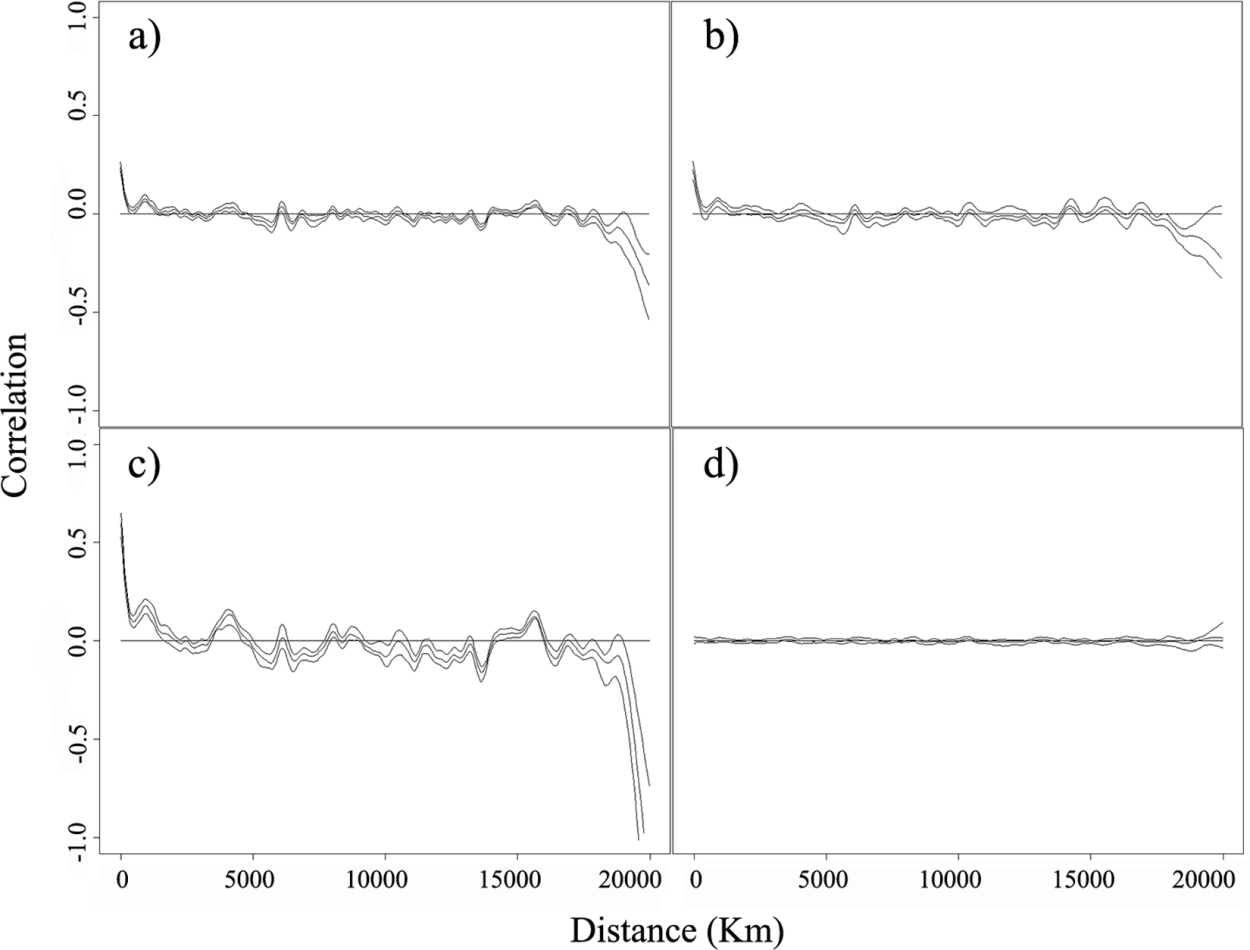
Correlograms of global amphibian chytrid fungus (*Batrachochytrium dendrobatidis*) data. *Batrachochytrium dendrobatidis* correlograms indicate positive spatial autocorrelation in chytrid presence between sampled sites: a) in full dataset; b) 50% sampled training data; c) predictions generated by the random forest model. However, spatial autocorrelation is absent in residuals from the random forest model (d).

Binary observations (*Bd* detection/non-detection) were positively spatially correlated (Fig 1A). We fitted a full model including all variables using both a logistic regression model and a general additive logistic regression model with a spline-based smoothed interaction term between longitude and latitude of the observations to see if model variables were capable of accounting for the spatial autocorrelation. However, residuals from both models remained spatially correlated. As a result, we opted for a nonparametric random forest classifier model to allow for implicit modeling of complex non-linear interactions. A random forest is a collection of random decision-tree classifiers that makes predictions by averaging the predictions of each individual decision tree model. Versions of the procedure differ in how randomness is introduced in the tree-building process. We implemented the procedure through scikit-learn in the Python environment [61], in which bootstrap samples of the original training data are used to train individual decision trees. A decision-tree learner is constructed recursively. At each step of the construction of the tree, a random set of variables is selected with equal probability from the suite of training variables. The learner then estimates how to optimally split the training examples into two nodes based on these selected variables so as to minimize the number of misclassified training points. The procedure is repeated until every node is pure (i.e., the node contains only positive or negative samples) or when some minimum number of observations for each node is reached.

We trained two sets of random forest models. First, we trained a forest on all the available data because latitude has been reported to be the most important control of climate [62] and sites were well-distributed along the latitudinal gradient. However, sampling effort was unequally distributed between geographic regions (S2 Table). To prevent an overall model from being overfitted to regions with larger sample sizes, we then trained separate forests for each region with more than 100 sites. Regions were downscaled geographic units, and were not intended to align with specific environmental conditions, *Bd* strains, or host taxa; regional results may provide insights for further hypothesis testing of these additional factors. Each forest contained 1000 trees with a maximum depth of 12, a minimum node size of 2, and specified that a maximum of 30% of all features may be used to split a node. These restrictions were imposed to prevent over-fitting of the trees to the training data. We randomly withheld 50% of the data as test data for cross-validation, and used the rest as training data for the forest (i.e., 2-fold or ‘holdout’ cross-validation). This procedure was repeated 100 times, with the evaluation metrics of mean accuracy and AUC (area under the receiver-operator curve) calculated to provide an estimate of prediction performance. Accuracy is defined as the percentage of trees in the forest that correctly classifies a site as *Bd*-detected or not detected, and mean accuracy is the average accuracy over all observations in the test set. Whereas each tree classifies an observation as 0/1 (detection/non-detection), the forest as a whole outputs the probability of *Bd* being detected for each site by averaging over all trees. To evaluate the performance of the forests, we need to select a probability cutoff point above which a site is classified as *Bd*-present. The true positive rate (the percentage of times that a *Bd*-positive site is correctly classified as present) and the false positive rate (the percentage of times that a *Bd*-negative site is incorrectly classified as positive) thus depend on the selection of the cutoff point. The receiver-operator curve is a curve defined by evaluating the true positive rate and false positive rate at variable cutoff points, and plotting the true positive rate against the false positive rate. The area under the curve (AUC) is maximized at 1 and will be large if at any cutoff point there is a desired large true positive rate and small false positive rate. A higher AUC indicates increased classification precision and is insensitive to the definition of an arbitrary cutoff point that separates negative from positive examples. The relative importance of a feature is evaluated by the decrease in classification accuracy when the values of that feature are permutated [54].

### Predictions

Using the fitted random forest models (trained with all data and trained separately for each region), we generated two sets of predictions of *Bd* occurrence probabilities based on current climatic conditions, as well as those predicted for the year 2100 by the Hadley Global Environment Model 2 –Atmosphere Ocean (HADGEM2—AO) with simulations from the International Panel on Climate Change (IPCC) Coupled Model Intercomparison Project Phase 5 (CMIP5). The HadleyGEM2 family of models are designed specifically for simulating centennial scale climate evolution [63], which aligns with the time window of our forecasts here. We predicted global *Bd* occurrence probabilities using the model trained with all data, and for regions with more than 100 sites using models trained specifically with data from those regions. Within the model, we chose the Representative Pathway Scenarios which are consistent with a wide range of climate change scenarios [63]. The RCPs are four greenhouse gas concentration trajectories adopted by the IPCC for its fifth assessment report, and describe four possible climate futures encompassing a wide range of possible future changes in anthropogenic greenhouse gas emissions–collectively they span the range of year 2100 radiative forcing values found in open literature [64]. These four RCPs were RCP2.6, RCP4.5, RCP6, and RCP8.5. The postfixes are chosen according to the increase in radiative forcing (the difference between solar energy absorbed by the Earth and that radiated back to space) in the year 2100 relative to pre-industrial values that each scenario describes (2.6, 4.5, 6.0, and 8.5 W∙ m^-2^, respectively, see Figure 10 in van Vuuren et al. [65] for detailed description and graphic representation. The four RCP scenarios correspond to increasingly severe warming scenarios and across all RCPs it is expected that global mean temperature will rise by 0.3 to 4.8°C by 2100 [65]. We report model results for scenarios 2.6, 6.0, and 8.5.

## Results

### Model validation and feature importance

**Fig 2:**
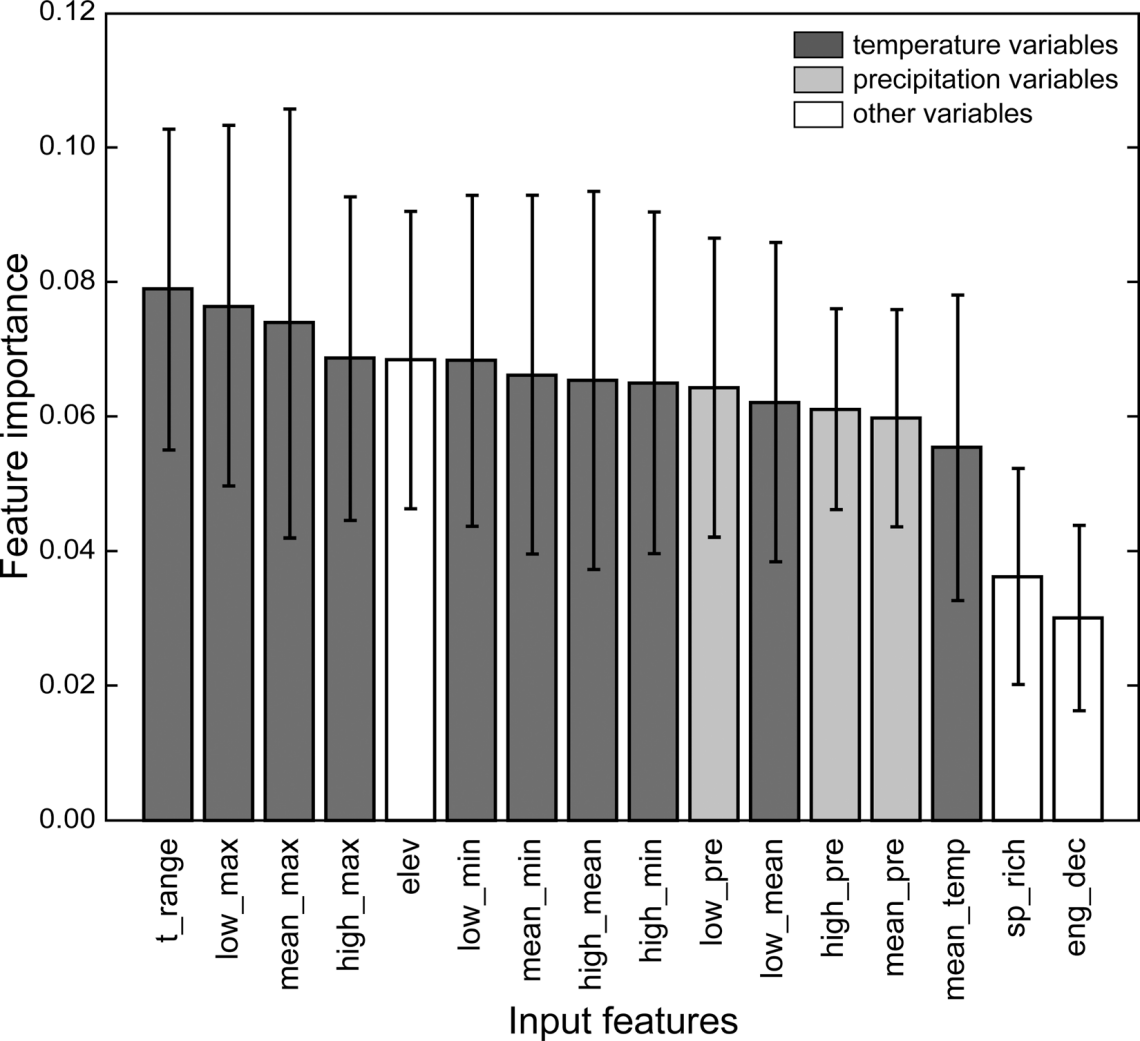
Relative importance of input features based on non-region specific (global) model of amphibian chytrid fungus (*Batrachochytrium dendrobatidis*) occurrence. Relative importance of an input feature for global *Batrachochytrium dendrobatidis* occurrence was estimated as an increase in out-of-bag error if training values for that feature were permutated. Plots indicate one standard deviation to show variation between trees in the random forest.

**Fig 3:**
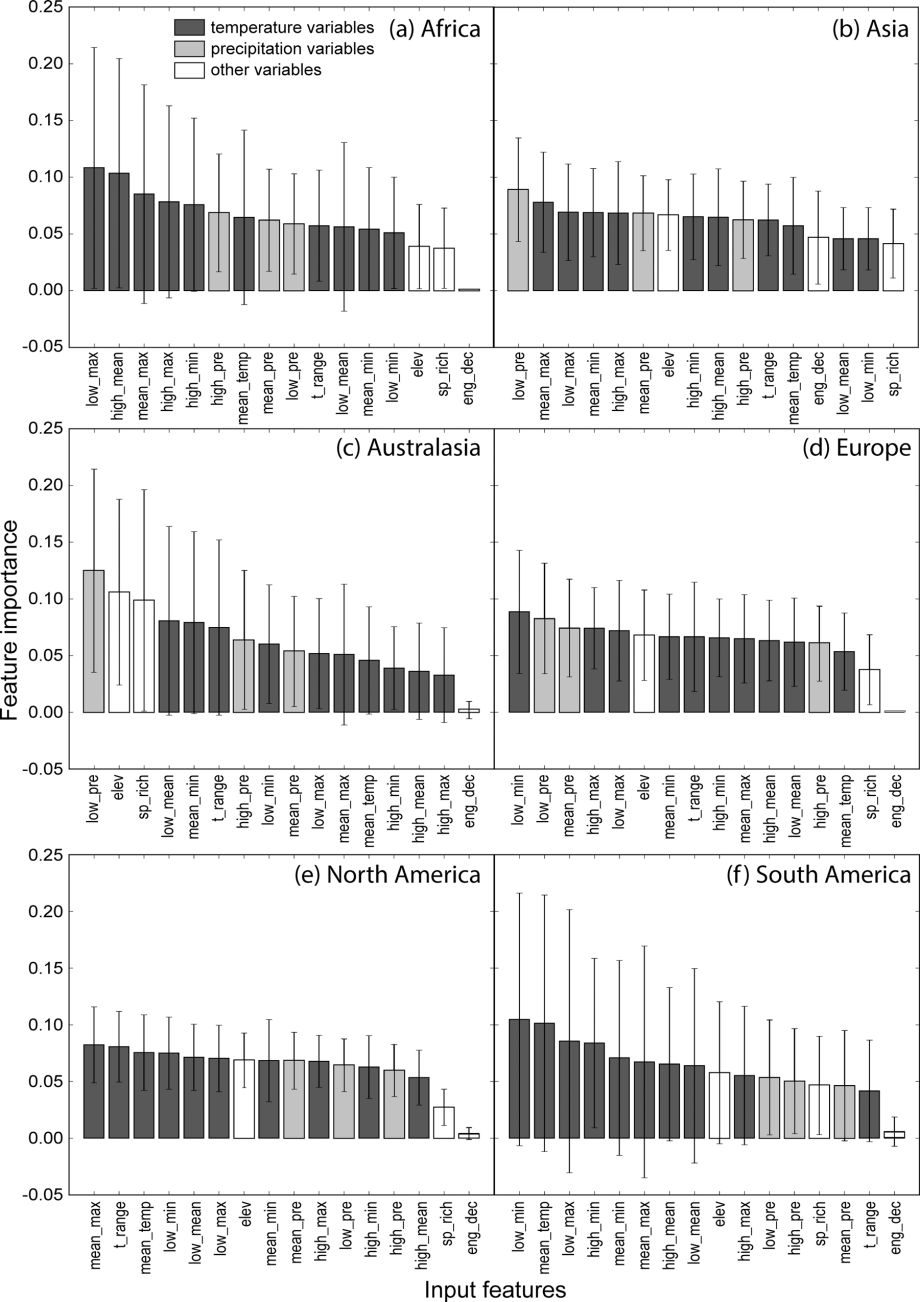
Relative importance of input features based on region-specific models of amphibian chytrid fungus (*Batrachochytrium dendrobatidis*) occurrence. Relative importance of input features for region-specific models of *Batrachochytrium dendrobatidis* occurrence were determined for: a) Africa; b) Asia; c) Australasia; d) Europe; e) North America; and f) South America.

For the random forest model fitted with all data, spatial autocorrelation remained in the 50% sampled training data and showed a trend similar to the full data set (Fig 1B). Predictions generated from the random forest models preserved the spatial autocorrelation structure, but residuals were not spatially correlated (Fig 1C–1D). These observations remained true for models fitted per region, and suggested that variables in the model and their interactions captured the spatial patterns in *Bd* distribution. Mean accuracy was 0.9071 (s.d. = 0.0046), and mean AUC was 0.9642 (s.d. = 0.0019) for the model trained with all data (S1 Table). AUC was uniformly high for all regions, but mean accuracy was lower in Australasia, Europe and North America. Although these three regions had the largest sample sizes, the model is not biased towards regions with fewer samples. However, region-specific models further improved performance both in terms of mean accuracy and AUC (S2 and S3 Tables). Model-based uncertainties in terms of the variation in predicted *Bd* occurrence probability per grid cell were low; the per-cell standard deviation ranged from 0.0024 to 0.087 for all models considered. In the model trained with all data, temperature range was the most important feature for classification. Temperature maxima were also important predictors, with the lowest, average and maximum monthly average maximum temperature ranked after temperature range. However, in general the input features do not differ substantially in terms of their relative importance (Fig 2). Region-specific models do not preserve the ordering of variable importance (Fig 3A–3F). Nonetheless, the importance of temperature and precipitation variables outweigh local amphibian species richness and whether a site has experienced enigmatic amphibian population declines (but see Asia as an exception where species richness seems to be at least as important as climate metrics; Fig 3B).

### Spatial patterns

**Fig 4:**
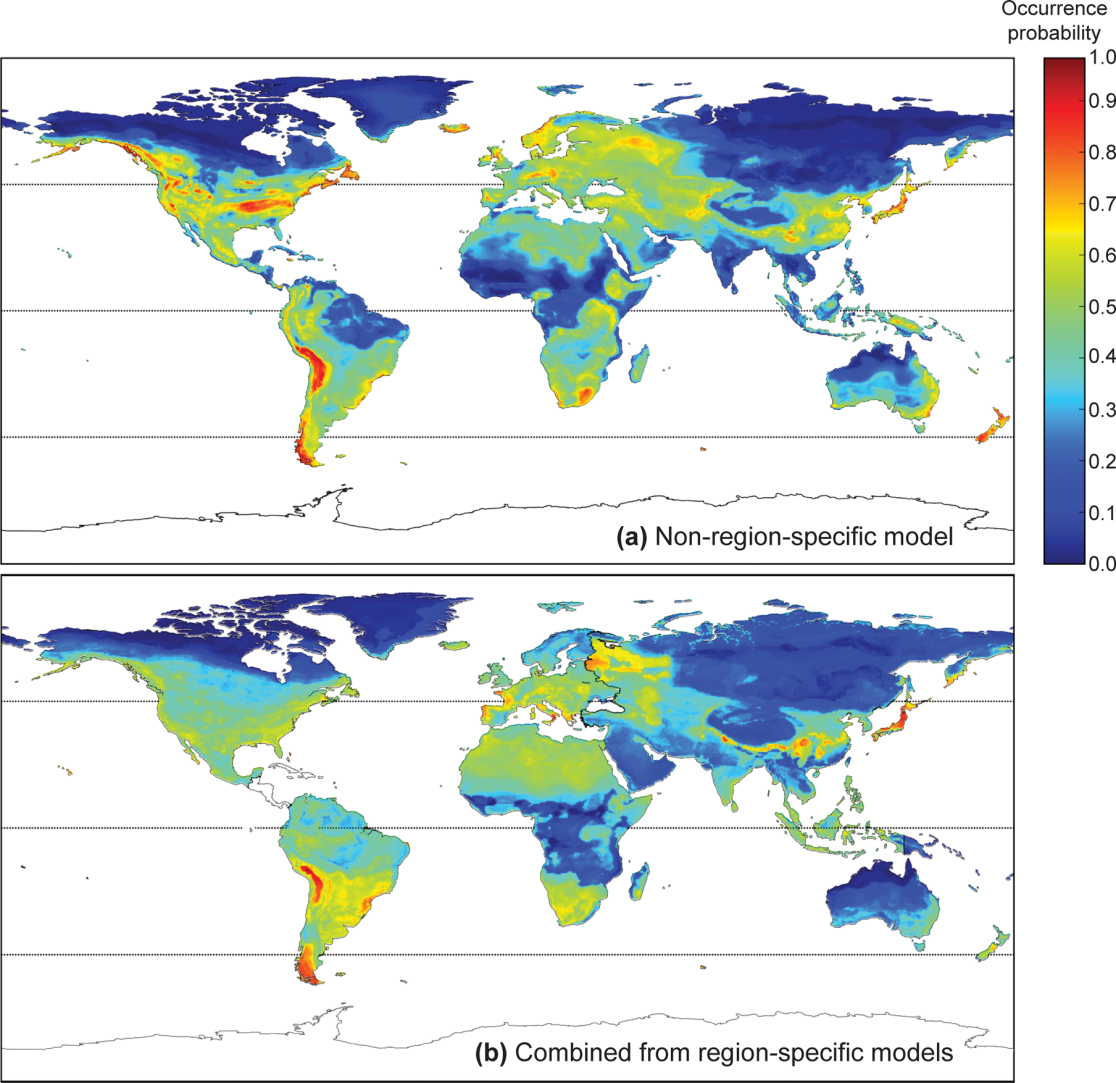
Predicted occurrence probability of amphibian chytrid fungus (*Batrachochytrium dendrobatidis*) with current climatic conditions. Predicted current occurrence of *Batrachochytrium dendrobatidis* based on: a) a non-region-specific global model; and b) data combined from region-specific models.

Similar to previous studies [38,42,43,45], the global (non-region-specific) model depicts that the areas of highest predicted *Bd* occurrence probabilities were patchily distributed (Fig 4A). These ‘hotspots’ occurred on all continents occupied by amphibians, but were more restricted in range in Eurasia, Oceania and Africa in contrast to the Americas. Around the equator, predicted *Bd* occurrence probabilities were generally low. In the Southern Hemisphere, south of the equatorial region, *Bd* occurrence probability generally increased, and hotspots were associated with coastal regions (e.g., Australia, Africa) and mountainous terrain of higher elevations (e.g., Andes in South America, Ethiopian highlands, and the highlands along the Great Rift Valley in Africa). In the Northern Hemisphere, where contiguous land masses extend further into the polar region, the higher latitudes in Asia and North America exhibited low predicted *Bd* occurrence probability. Hotspots were again associated with areas of high elevation (e.g., the Kunlun, Qinling, Taihang, and Nanling mountains in China, Hida and Ou mountains in Japan, the Rockies, Appalachians, and Interior Highlands of the south-central United States in North America). In Europe, hotspots coincided with the Alps, the Kjolen and Ural mountains.

Based on region-specific models compiled for a global perspective, the distribution of areas with higher *Bd* occurrence probability largely coincided with those predicted by the non-region-specific global model (Fig 4B). However, there was a decrease in overall predicted *Bd* occurrence probabilities, and this trend was especially true for Asia and Australasia. Areas representing low to intermediate (0 to 0.6) *Bd*-occurrence probability predicted by regional models did not fully agree with those from the global model. In North America, the region-specific model predicted no apparent ‘hotspots’ in the mid-latitudes. In the higher latitudes the same model predicted that the region of intermediate *Bd* occurrence probabilities would extend further into the Arctic than the global model. In contrast, in Asia and Australasia, region-specific models predict that the areas of low *Bd* occurrence probability (predominantly in the higher latitudes) would extend further into the mid-latitudes in comparison to the global model. In South America, the most disagreement occurred in the northern regions. The region-specific model predicted that the northern Andes did not contain noticeable hotspots, and that the Amazon basin would have intermediate *Bd* occurrence probabilities. In contrast, the global model predicted an area of low *Bd* occurrence in the Amazon basin and hotspots in the northern Andes (note, however, that the training data for the models contained no sites from the interior Amazon basin). In Africa, regions of low *Bd* occurrence probabilities (0 to 0.2) were shifted more toward the equator in the region-specific model. These differences suggest that while maps may be used to identify *Bd* hotspots (which are largely in agreement), caution needs to be applied in interpreting spatial risk of *Bd* where models predict low to intermediate occurrence probability.

**Fig 5:**
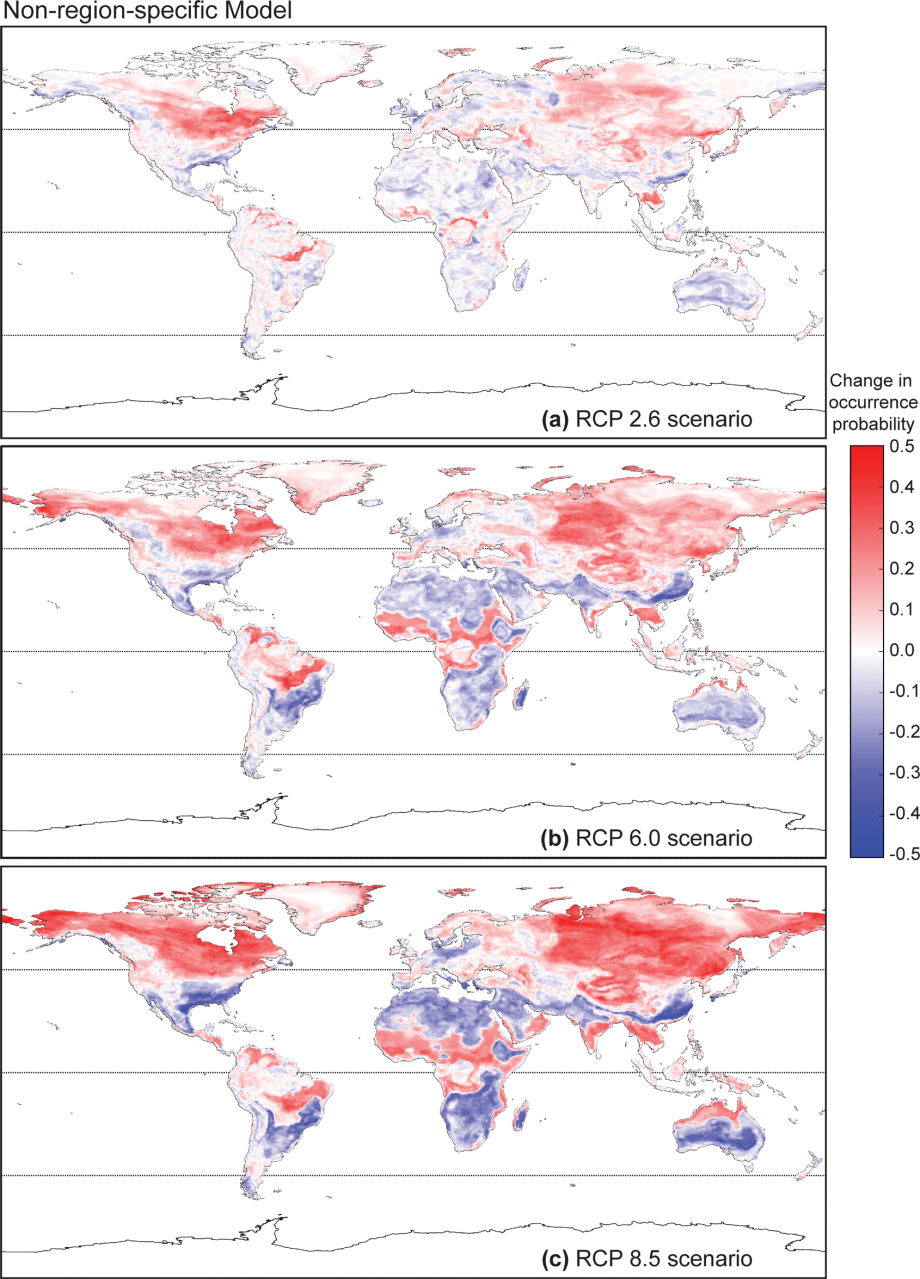
Predicted change in occurrence probability of amphibian chytrid fungus (*Batrachochytrium dendrobatidis*) based on future climate conditions using a non-region-specific global model. Predicted change in occurrence probability of *Batrachochytrium dendrobatidis* determined using the non-region-specific global model with future climate conditions forecasted for 2100 using RCP 2.6, 6.0 and 8.5 scenarios.

The relative changes in predicted *Bd* occurrence probability in the year 2100 as predicted by the non-region-specific global model differ across three RCP scenarios (2.6, 6.0, 8.5; Fig 5A–5C). The area and extent of relative change become larger with projected increases in radiative forcing (see methods for definition), showing both increases and decreases in *Bd*-occurrence probabilities among regions. Changes are most notable in the higher latitudes of the northern hemisphere, where large areas in North America and Asia that were previously unsuitable for *Bd* exhibited the largest increases in predicted *Bd* occurrence probability. This area of increase shifted progressively north with an increase in radiative forcing. Other areas of increase include forested areas around the equator such as the Malaysian and Indonesian islands, northeastern Brazil, and lowland regions around the equatorial belt in Africa. The high-elevation regions that were previously associated with predicted *Bd* occurrence hotspots generally exhibited shrinkages, which is particularly apparent in the Andes Mountains of South America. Due to the sensitivity of the *Bd* fungus to temperature and the apparent importance of temperature as a predictor variable, it may be that climate change shifts these high-altitude regions out of the optimum thermal envelope for *Bd* [66].

**Fig 6:**
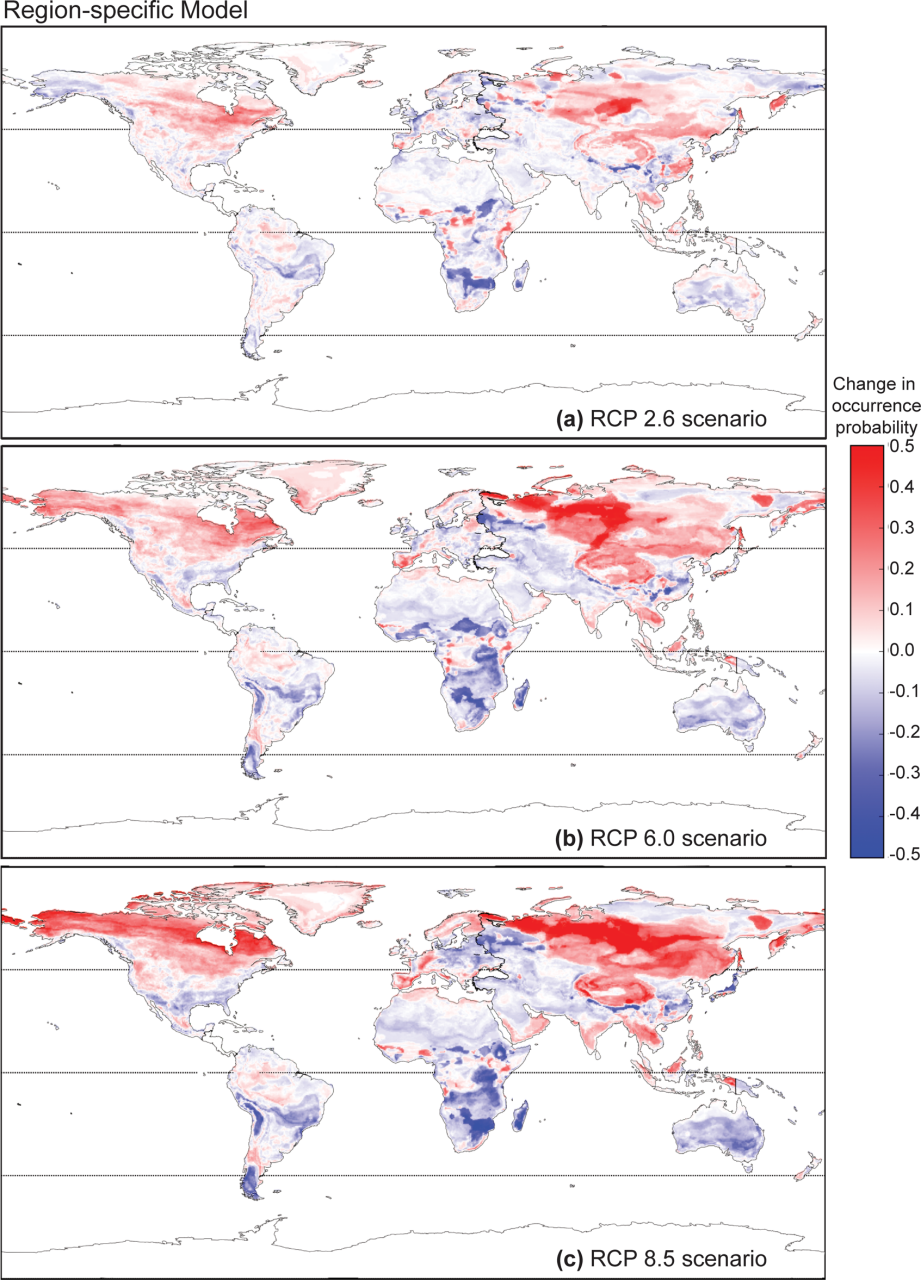
Predicted change in occurrence probability of amphibian chytrid fungus (*Batrachochytrium dendrobatidis*) based on future climate conditions using data combined from region-specific models. Predicted change in occurrence probability of *Batrachochytrium dendrobatidis* determined using data combined from region-specific global models with future climate conditions forecasted for 2100 in the RCP 2.6, 6.0 and 8.5 scenarios.Predicted future occurrence of *Batrachochytrium dendrobatidis* determined using the non-region-specific global model based on future climate conditions forecasted for 2100 in the RCP 2.6, 6.0 and 8.5 scenarios. Predicted future occurrence of *Batrachochytrium dendrobatidis* determined using the non-region-specific global model based on future climate conditions forecasted for 2100 in the RCP 2.6, 6.0 and 8.5 scenarios.

This trend of more drastic change associated with increased radiative forcing remained true for predictions generated by the region-specific models (Fig 6A–6C). Under climate change projected by RCP 2.6, the regions of relative increase and decrease approximately agree with the global model for most geographic regions with the exception of Australasia. In this case, the region-specific model predicted that continental Australia will undergo a decrease in overall *Bd-*occurrence probability in the coastal regions and an increase inland. The global model, however, predicted the opposite. Under RCP 6.0, a major inconsistency lies in predictions close to the equatorial regions. The global model predicted an increase in *Bd* occurrence probability in the Amazon basin and other forested regions near the equator in Africa and the Indonesian and Malaysian islands. This increase was much less prominent according to the region-specific models. Again, Australasia stands out in that while the region-specific models predict overall no change or a decrease in *Bd* occurrence probability, the global model predicts an increase in coastal regions. The differences in predicted *Bd* occurrence probabilities under RCP 8.5 between region-specific and non-region specific models were similar to those observed under RCP 6.0. Despite these differences, all models and climate scenarios were consistent in predicting an increase in *Bd* occurrence probabilities in the higher latitudes, with the zones of highest increase shifting progressively northward further into the Arctic with increases in radiative forcing.

## Discussion

As with free-living species, pathogens likewise have optimum climatic envelopes and deviations from the optimum may make them susceptible to potential distribution shifts in the presence of climate change [67]. It has been argued that since *Bd* prefers cool, moist conditions, its emergence might be curbed by climate warming [44]. However, as is consistent with observed responses to ongoing climate change in other species [4,68], our models generally forecast that *Bd* occurrence probabilities will experience the greatest increase in the higher-latitude regions of the Northern Hemisphere, which may correspond to a range shift. It is unclear whether this range shift will represent a shrinkage or expansion in overall *Bd* distribution, since the larger landmass at high latitudes in the Northern Hemisphere may compensate for lost range in the lower latitudes.

This increase in *Bd*-occurrence probability in the northern temperate zones due to climate change has been previously suggested by Bosch et al. [39], who showed that the occurrence of *Bd*-related disease was linked to rising temperatures that moderated severe winters. This non-uniformity in projected degree of *Bd* environmental suitability changes may be reflective of the non-uniformity in the rate of climate change at different locales. Between years 1948 and 2010, the strongest rates of spring and winter warming have been observed to occur more frequently at latitudes of 30 to 90° N [69]. As a consequence, temperature seasonality in the Northern Hemisphere has diminished over time at mid to high latitudes from 30 to 90° N [60], which may account for why we observed a prominent increase in projected *Bd* environmental suitability in these regions. Conversely, the decreases in *Bd* environmental suitability in the tropical regions projected by our model may be due to temperature seasonality being amplified in some tropical regions, including in Africa and the Middle East [69].

In addition, changes in precipitation are expected to scale approximately linearly with increasing warming and is generally expected to increase in higher latitudes [70–72], which may further enhance the environmental suitability of the temperate zones to *Bd*. Observations of current climate shifts indicate that increases in precipitation over Northern Hemisphere mid- and high latitude land areas have a strong correlation to increases in total cloud amount due to temperature increases inducing evaporation [73]. In contrast to the Northern Hemisphere, no comparable systematic changes have been detected in broad latitudinal precipitation averages over the Southern Hemisphere [73]. This also may potentially account for the differences in the magnitude of environmental suitability increase between the Northern and the Southern Hemisphere we observed from our projections.

However, these broad descriptions may be difficult to generalize as long-term rainfall has actually been observed to decrease in some large regions including the Mediterranean and parts of southwestern North America [74,75]. Furthermore, the confidence of precipitation projections remains lacking in many parts of the globe and at smaller spatial scales, and previous projections of hydroclimate has been shown to be erroneous [76,77]. This adds another source of uncertainty in our *Bd* occurrence projections, which is especially concerning since our models suggest that *Bd* occurrence is not dominantly dependent on temperature alone and that precipitation and its interactions with temperature might be equally important.

Our region-specific models possessed higher predictive performance than our global model, and generated predictions that are consistent with some previous SDMs [22,38,41–43,45]. Both our region-specific and global models predicted that the extent and magnitude of change in *Bd* occurrence probabilities increased with projected changes in radiative forcing relative to preindustrial levels. In general, more extensive areas of larger increases in predicted *Bd* occurrence probability are expected with increasing radiative forcing.

Yet, inconsistencies exist in the predictions generated by the region-specific and global models. Under all climate-future scenarios, the non-region-specific model predicted a higher overall *Bd* occurrence probability. These mismatches are especially noticeable in regions of the Southern Hemisphere, with more substantial disagreement between models in equatorial regions and continental Australia. This suggests that the *Bd*-climate relationship differs among regions, which may be due to variation in the *Bd* strain among regions [78], heterogeneous *Bd* ecology, host-*Bd* dynamics, or other unmeasured variables; these hypotheses warrant consideration in subsequent studies, as our intent for regional analyses here was to simply downscale the scope of the analysis to geographically relevant units. However, our region designations are purely geographical and may not necessarily correspond to biological meaningful subunits of the *Bd* lineage. Therefore the differences observed between regional models could also have resulted from each model describing only the partial niche instead of the full niche of *Bd*. Specific to the Malaysian and Indonesian islands, northeastern Brazil, and lowland regions around the equatorial belt in Africa, we found the predictions from our global model also were inconsistent with projections obtained by Rödder et al. [45]. Both our region-specific and global models agreed with Rödder et al. [45] in that, based on present conditions, *Bd* occurrence probability should generally be low in these regions. This is in-line with the empirical observation that under laboratory settings, higher temperatures inhibit the growth of *Bd* and in extreme cases, induce mortality [28]. However, our global model predicted a general increase in *Bd* occurrence probabilities in these regions (equatorial zones) while Rödder et al. [45] forecasted little change or a decrease. We suspect that these model uncertainties in the tropical zone are due to extrapolations outside of the climate data range in the available *Bd* records we used as the training set for our models.

Under climate change, novel climatic conditions are predicted extensively. However, in the tropics, forecasted climate conditions fall outside of the observed present range and therefore are “novel” to the prediction model since they are unobserved in the training data, which produces model uncertainties due to extrapolation. Whereas even as conditions in non-tropic zone change and novel climate conditions are experienced locally, the precise temperature and precipitation values are still contained within the range of the present training data and therefore predictions generated for these regions are more akin to interpolation. Currently, the tropics already experience the highest temperature and precipitation regimes. Even if aggressive measures were taken to reduce greenhouse gas emissions, the annual mean temperature in an average location likely would be shifted out of its previous normal range due to climate change by 2069 [79]. Therefore, predictions generated for this region beyond this time frame will likely be based on extrapolating outside the range of climatic variables present in the training data. As a result, while the tropics are affected by climate change to a lesser degree than temperate zones [79], model uncertainties in these regions warrant further investigation and monitoring of *Bd* presence.

In addition to these model uncertainties, our prediction maps are point estimates only at a coarse spatial scale and do not reflect microhabitat, seasonal, or annual variations in climate; therefore at finer spatial and temporal scales they may not be fully reflective of the relative spatial risk of *Bd* presence. Furthermore, complex local climate change dynamics between temperature and precipitation may not be adequately reflected in the present collection of variables used in our model. In addition to using static estimates of future climate metrics, the velocity of climate change itself may be incorporated into the models as an additional measure of how future *Bd* distribution may adapt to local environments [80]. Another finer-scale consideration that could be addressed in additional down-scaled models is habitat change and fragmentation that could affect both host dispersal and *Bd* transmission to changing climate niches across landscapes [30]. Despite an accruing amount of knowledge about *Bd* physiology in laboratory settings, our study illustrates that it might be too simplistic to assume that global warming will curtail the spread of *Bd*. Furthermore, the association between environmental temperature and amphibian immunity [81,82] could confound how local populations might fare under climate change, as changing temperature and precipitation regimes may act to simultaneously increase amphibian susceptibility to *Bd* [48,83].

Much focus of how climate change will affect *Bd* and chytridiomycosis outbreaks has been on the role of temperature, one of the most important environmental factors that influences *Bd* growth and development of chytridiomycosis [84]. Intuitively, it would seem that increasing temperatures might be expected to decrease *Bd* occurrence probabilities due to the fungus’ intolerance to high temperatures in vitro [28]. However, *Bd*-associated amphibian population declines in the Neotropics were more likely to occur after high-temperature years [50], suggesting that climate change might actually increase *Bd* occurrence probabilities [46]. Moreover, other factors may interact with those associated with climate change to influence amphibian population declines [85,86].

Although we cannot extend causal inference from observation data, our analysis nonetheless provide support for the hypothesis that climate change might further promote the emergence of *Bd*, especially in the temperate zones. While this further highlights the controversy and the need for research on *Bd*-climate relationships outside of laboratory settings, we note that in contrast to previous *Bd* SDMs that relied on additive regression models (e.g., MaxEnt), our model identifies no single climatic variable as wholly determining *Bd* occurrence probabilities as none of the main variables held overwhelming predictive power. Therefore, it is prudent that research attention on *Bd*-climate relationships should not be limited to temperature regimes alone; rather, there is support for considering complex interactions of climatic variables. Moreover, since *Bd* sampling is predominantly conducted by swabbing amphibians and *Bd* may be found outside of amphibian hosts [35], it is possible that sites where *Bd* has not been detected from host samples, currently deemed *Bd*-negative in our analyses, might underrepresent actual *Bd* occurrences and therefore our model may be biased downwards in representing actual *Bd* occurrence probabilities. Although this can only be remedied by more sampling for *Bd*, including broader environmental sampling, here we caution that SDMs based on these data may be systematically under-predicting *Bd* occurrence probabilities and that our predictions may be also be influenced by sampling bias towards locations that *a priori* are deemed suitable for *Bd* and certainly biased towards sampling within amphibian populations.

Our models had higher performances than previous efforts, yet it is important to again emphasize the correlative nature of SDMs in general. The model we presented here is a non-parametric random forest learning procedure capable of implicitly including complex higher-order interactions. It is possible that the climate variables we have chosen to include here and their interactions yield excellent predictive performance simply because they are highly correlated with other unmeasured variables at each site, which may in fact be the driver of *Bd* presence. While temperature and moisture are certainly important variables that determine the propagation of *Bd* on the host and in the environment, we should exercise caution in inferring causation from correlative models based on predictive performance. We acknowledge that future predictions from our SDM will rest on the assumption of constant relationship between *Bd* occurrence and climate variables, and does not consider the effects of coevolution or local adaptations. We further point out here that while our model aims to predict the environmental suitability to *Bd* as a proxy of its occurrence probability, occurrence at a site may not necessarily be equivalent to *Bd* having a significant impact at the local population (i.e., local declines or extinctions) and will depend on specific host-*Bd* interactions.

## Conclusions

Using the most comprehensive set of *Bd* surveillance data available, we implemented a machine-learning model that uses both detection and non-detection data to predict current and future distributions of *Bd* under climate change scenarios based on different levels of forecasts in the amount of anthropogenic greenhouse emissions. We built random forest models combining data gathered from all regions in the world (non-region-specific “global” models), and also models using region-specific data from relatively well-sampled geographic regions (minimum of 100 observations per region, actual regions entering the analysis contained at least 221 observations.). We found that by using climate variables, we captured the spatial correlation patterns of the current *Bd* distribution. This observation supports climate variables as strongly influencing the present distribution of *Bd*. Using future climate projections under several IPCC scenarios, our models predicted that *Bd* ranges will shift into higher latitudes and altitudes, especially in the temperate zones of the Northern Hemisphere. Our projections are useful for the development of monitoring designs in these areas, especially for sensitive species and those vulnerable to multiple threats.

## Supporting Information

S1 Fig Global site records for the amphibian chytrid fungus, *Batrachochytrium dendrobatidis*. (TIF)

S1 Table Input features in the random forest models and their definitions. These values were extracted for each coordinate for each year during 2000–2010, then averaged over the period.
(PDF)

S2 Table Regional samples sizes (sites per region) for the amphibian chytrid fungus (*Batrachochytrium dendrobatidis*), chytrid occurrence probabilities, and validation results based on random forest models trained on combined worldwide data. AUC = area under the receiver-operator curve.
(PDF)

S3 Table Mean accuracy and area under the receiver-operator curve (AUC) for each region based on separate random forest models trained on amphibian chytrid fungus (*Batrachochytrium dendrobatidis*) data from each region. (PDF)
