# Supplementary figures and images for: Projecting the Global Distribution of the Emerging Amphibian Fungal Pathogen, Batrachochytrium dendrobatidis, Based on IPCC Climate Futures
Source: PLoS One. 2016 Aug 11;11(8):e0160746. doi: 10.1371/journal.pone.0160746 (PMC4981458; doi:10.1371/journal.pone.0160746)

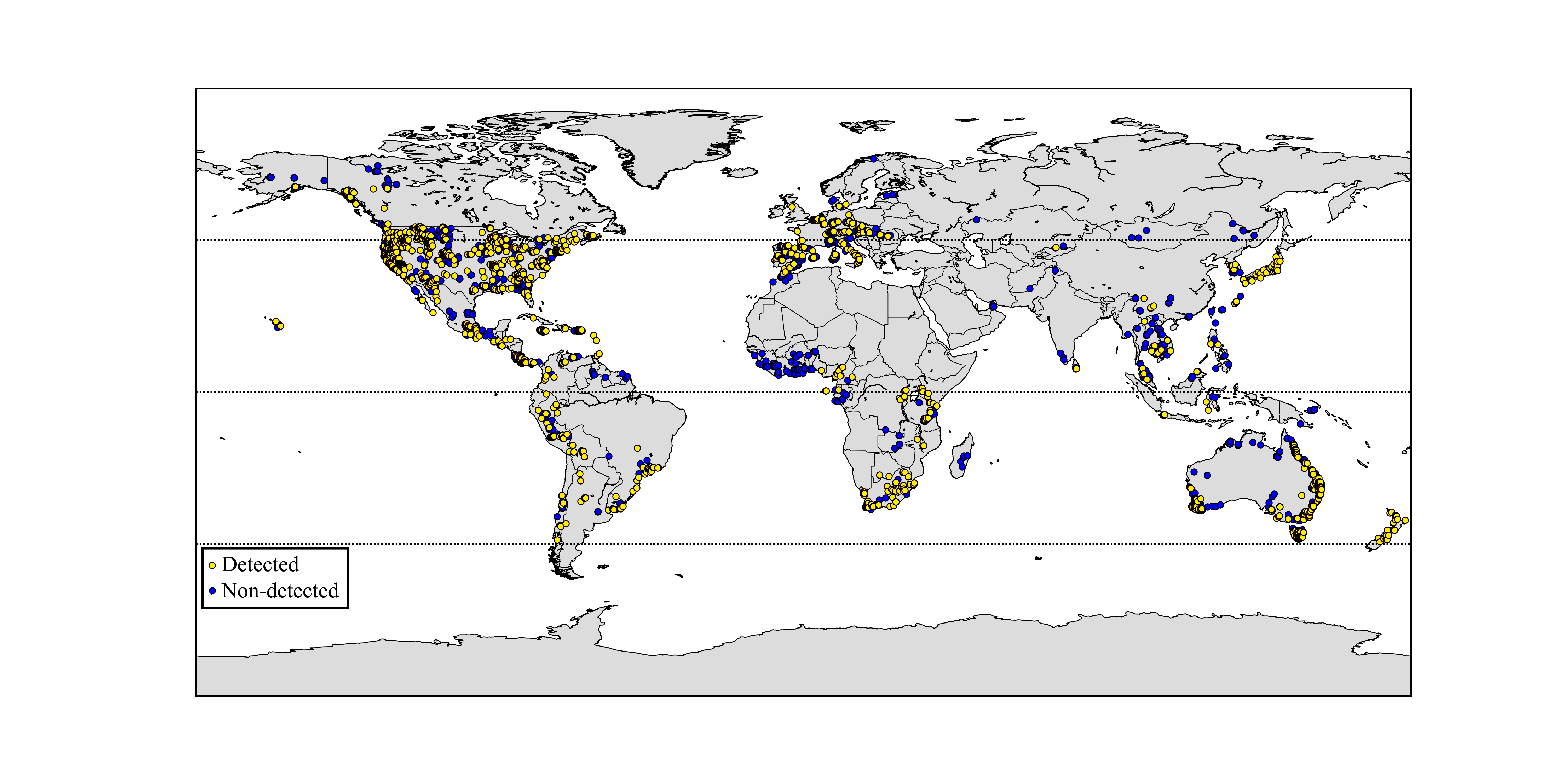

Supplement: S1 Fig — (TIF) [file pone.0160746.s001.tif]
