# Supplementary material for: Projecting the Global Distribution of the Emerging Amphibian Fungal Pathogen, Batrachochytrium dendrobatidis, Based on IPCC Climate Futures
Source: PLoS One. 2016 Aug 11;11(8):e0160746. doi: 10.1371/journal.pone.0160746 (PMC4981458; doi:10.1371/journal.pone.0160746)
Supplement: S1 Table — These values were extracted for each coordinate for each year during 2000–2010, then averaged over the period. (PDF) [file pone.0160746.s002.pdf]

S1 Table. **Input features in the random forest models and their definitions.** Values were extracted for each coordinate for each year during 2000–2010, then averaged over the period.

Variable name	Definition	Units
**mean_temp**	mean annual temperature	°C
**high_mean**	highest monthly average temperature in a year	°C
**low_mean**	lowest monthly average temperature in a year	°C
**mean_max**	annual mean of mean monthly maximum daily temperature	°C
**high_max**	annual maximum of mean monthly maximum daily temperature	°C
**low_max**	annual minimum of mean monthly maximum daily temperature	°C
**mean_min**	annual mean of mean monthly minimum daily temperature	°C
**high_min**	annual maximum of mean monthly minimum daily temperature	°C
**low_min**	annual minimum of mean monthly minimum daily temperature	°C
**t_range**	high_max – low_min	°C
**mean_pre**	annual mean of monthly precipitation	mm
**high_pre**	annual maximum of monthly precipitation	mm
**low_pre**	annual minimum of monthly precipitation	mm
**elevation**	the altitude of a site	m
**eng_dec_in**	whether the site has documented enigmatic amphibian declines	0/1
**sp_rich**	estimated number of amphibian species at the site	[number]
**biome**	biome designation at site	[categorical type]
