# Supplementary material for: Projecting the Global Distribution of the Emerging Amphibian Fungal Pathogen, Batrachochytrium dendrobatidis, Based on IPCC Climate Futures
Source: PLoS One. 2016 Aug 11;11(8):e0160746. doi: 10.1371/journal.pone.0160746 (PMC4981458; doi:10.1371/journal.pone.0160746)
Supplement: S2 Table — AUC = area under the receiver-operator curve. (PDF) [file pone.0160746.s003.pdf]

S2 Table. Regional samples sizes (sites per region) for the amphibian chytrid fungus (*Batrachochytrium dendrobatidis, Bd*), chytrid occurrence probabilities, and validation results based on random forest models trained on combined worldwide data. AUC = area under the receiver-operator curve.

Region	Sample size (no. sites)	% *Bd*-detected sites	Mean accuracy	AUC
**Africa**	333	0.3544	0.9550	0.9886
**Asia**	419	0.1718	0.9761	0.9981
**Australasia**	847	0.4451	0.8737	0.9488
**Caribbean**	28	0.6429	1.0000	1.0000
**Central America**	27	0.6296	0.9630	1.0000
**Europe**	770	0.3039	0.8623	0.9458
**North America**	2333	0.4483	0.8933	0.9635
**South America**	210	0.5429	0.9429	0.9938
