# Supplementary material for: Projecting the Global Distribution of the Emerging Amphibian Fungal Pathogen, Batrachochytrium dendrobatidis, Based on IPCC Climate Futures
Source: PLoS One. 2016 Aug 11;11(8):e0160746. doi: 10.1371/journal.pone.0160746 (PMC4981458; doi:10.1371/journal.pone.0160746)
Supplement: S3 Table — (PDF) [file pone.0160746.s004.pdf]

S3 Table. Mean accuracy and area under the receiver-operator curve (AUC) for each region based on separate random forest models trained on amphibian chytrid fungus (*Batrachochytrium dendrobatidis*) data from each region.

Region	Mean accuracy	Standard deviation (mean accuracy)	AUC	s.d. (AUC)
**Africa**	0.998126	0.001111	0.976451	0.009383
**Asia**	0.999541	0.000468	0.983954	0.007389
**Australasia**	0.99115	0.002234	0.950904	0.008226
**Europe**	0.997332	0.001014	0.965998	0.006929
**North America**	0.977234	0.002675	0.914089	0.006598
**South America**	0.99725	0.001883	0.97518	0.012991
